# Exosomes Derived from Nerve Stem Cells Loaded with FTY720 Promote the Recovery after Spinal Cord Injury in Rats by PTEN/AKT Signal Pathway

**DOI:** 10.1155/2021/8100298

**Published:** 2021-07-14

**Authors:** Jianbin Chen, Can Zhang, Shouye Li, Zheming Li, Xiaojing Lai, Qingqing Xia

**Affiliations:** ^1^Department of Neurosurgery, Tongji Hospital, Tongji Medical College, Huazhong University of Science and Technology, Wuhan, Hubei 430030, China; ^2^Department of Biomedical Engineering, College of Biology, Hunan University, Changsha 410082, China; ^3^College of Pharmacy, Hangzhou Medical College, Hangzhou, 310022 Zhejiang, China; ^4^The Cancer Hospital of the University of Chinese Academy of Sciences (Zhejiang Cancer Hospital), Hangzhou, 310022 Zhejiang, China; ^5^Institute of Basic Medicine and Cancer (IBMC), Chinese Academy of Sciences, Banshan East Road 1, Gongshu District, Hangzhou, 310022 Zhejiang, China; ^6^Department of Laboratory Medicine, Huangyan Hospital of Wenzhou Medical University (Taizhou First People's Hospital), Hengjie Road 218, Huangyan District, Taizhou, 318020 Zhejiang, China

## Abstract

**Background:**

Spinal cord injury (SCI) remains a challenge owing to limited therapies. The exosome of neural stem cells (NSCs-Exos) and FTY720 transplantation could improve SCI effectively. However, the effect and mechanism of NSCs-Exos combined with FTY720 (FTY720-NSCs-Exos) transplantation in the treatment of SCI are not fully understood.

**Methods:**

Sprague Dawley rats (8-week-old) were used to establish the SCI model, followed by the treatment of NSCs-Exos, FTY720, and FTY720-NSCs-Exos. The effect of FTY720, NSCs-Exos, and FTY720-NSCs-Exos combination treatment on hindlimb function, pathological changes, apoptosis activity, and the expression of spinal edema-related proteins and apoptosis-related proteins in SCI models were investigated by BBB scoring, HE staining, TUNEL staining and immunohistochemistry, and Western blotting. Meanwhile, the effect of these treatments on spinal cord microvascular endothelial cells (SCMECs) was detected under hypoxic circumstance.

**Results:**

Our results found that FTY720-NSCs-Exos could alleviate pathological alterations and ameliorate the hindlimb function and oxygen insufficiency in model mice after SCI. In addition, exosomes could ameliorate the morphology of neurons, reduce inflammatory infiltration and edema, decrease the expression of Bax and AQP-4, upregulate the expression of claudin-5 and Bcl-2, and inhibit cell apoptosis. At the same time, *in vitro* experiments showed that FTY720-NSCs-Exos could protect the barrier of SCMECs under hypoxic circumstance, and the mechanism is related to PTEN/AKT pathway.

**Conclusion:**

FTY720-NSCs-Exos therapy displayed a positive therapeutic effect on SCI by regulating PTEN/AKT pathway and offered a new therapy for SCI.

## 1. Introduction

Spinal cord injury (SCI) is a common and harmful injury in spinal surgery with 0.6–0.9 million new patients worldwide every year [1]. Symptoms of SCI range from minimal dysfunction to quadriplegia and consist entirely of sensory, motor, and autonomic dysfunction [2]. Usually, after the primary traumatic mechanical destruction of the spinal cord tissues, secondary damage was rapidly triggered, such as inflammation, hypoxia, ischemia, and neuronal apoptosis [3]. Over time, SCI will eventually lead to motor and sensory dysfunction [4]. However, improving the recovery of neurological function after SCI is still the biggest challenge.

Nowadays, stem cell transplantation is increasingly used for SCI therapy. This may be due to its pluripotency and can be induced into a variety of cells [5]. Several studies have showed that transplantation of neural stem cells (NSCs) can ameliorate the function of motor nerve, sensory, and/or autonomic nerve in SCI [6–8]. However, stem cell transplantation has lots of disadvantages, such as low survival rate, immune rejection, dedifferentiation, and malignant tumor formation according to previous studies [9, 10]. At present, studies have shown that the efficacy of NSCs is mainly due to the role of their secreted exosomes [11, 12]. As the crucial paracrine factors of stem cells, the diameter of exosomes was between 20 and 150 nm and composed of a lipid bilayer that encapsulates RNA, DNA, and soluble proteins [13, 14]. Several studies have shown that exosomes of NSCs can protect neuronal function, promote neurocognitive impairment, and SCI repair [8, 15, 16]. It is used to treat liver, cardiovascular, and kidney injuries.

FTY720 is a functional antagonist of sphingosine 1-phosphate receptor-1 (S1P1), has a longer half-life *in vivo*, and can play an immunomodulatory function [17]. It has been proved that the systemic application of FTY720 has a positive effect on the recovery of SCI [18, 19]. However, it may cause serious adverse effects by systemic administration of FTY720. The locally released FTY720 is more conducive to remodel microvascular and regenerate bone tissue [20]. Therefore, we used the exosomes of NSCs (NSCs-Exos) as the carrier of FTY720 to detect the repairment of SCI, including behavioral assessment, inflammatory response, neuronal apoptosis, and spinal cord edema. Meanwhile, the improvement of FTY720-NSCs-Exos on SCMECs under hypoxic condition and its potential mechanism were also discussed. This study showed that localized delivery of FTY720 can ameliorate the injury of SCI through PTEN/AKT signaling pathway and offer a new therapeutic method for SCI.

## 2. Materials and Methods

### 2.1. NSCs-Exos Generation and Collection

Rat Fetal NSCs (N7744100, USA) were purchased from Thermo Fisher Scientific. Before NSC culturing, fetal bovine serum was centrifuged at 110000 g for 6 h to remove FBS's own exosomes. Then, the NSCs were cultured in a medium which contained FBS for 48 h when it reached 60–80% confluence. Then, the supernatant of the medium was collected, and separated exosomes by multistep centrifugation according to the previous studies [21, 22]. In brief, the supernatant was centrifuged at 300 g for 10 min, 1000 g for 20 min, and 10000 g for 30 min to remove the debris and dead cells. After filtering with a 0.22 *μ*m filter membrane, the supernatant was centrifugated at 110000 g for 70 min to remove contaminated proteins. After filtering with a 0.22 *μ*m filter membrane, the exosomes were saved at -80°C. Meanwhile, CD9 and CD81 were detected by Western blotting.

### 2.2. Characterization of NSCs-Exos

NSCs-Exos were fixed with 2% paraformaldehyde (PFA) and loaded to 200-mesh copper grids. After fixing with 1% (*w*/*v*) glutaraldehyde, the grids were stained with 4% uranyl acetate, then washed and dried in darkness at room temperature. After that, the grids were observed by Transmission Electron Microscopy (TEM, HT-7700,120 KV) to detect the morphology of NSCs-Exos.

The size of NSCs-Exos was detected by nanoparticle-tracking analysis (NTA) according to previous studies [23, 24]. The NSCs-Exos were diluted in PBS (1 : 10) and recorded via videos (30 s for each time, three times). Then, NTA sofware (N30E, NanoFCM) was performed to analyze the particle size.

### 2.3. Exosome Labeling with FTY720

The purifed NSCs-Exos (3 mg) was dispersed in PBS solution (0.9 mL). Then, 0.1 mL FTY720 DMSO solution was added and sonicated 15 cycles (2 seconds per cycle, stop for 2 seconds) in an ultrasonic cell pulverizer. After that, the mixture was incubated at 37°C for 60 minutes. Centrifugation (12000 g, 30 minutes) was performed to get rid of unbound FTY720. After that, the FTY720-NSC-Exos pellet was washed 3 times and resuspended in PBS to an extent concentration. After passed through 0.22 *μ*m syringe filter, FTY720-NSC-Exos was used for further study.

### 2.4. Detected the Concentration of FTY720 in FTY720-NSC-Exos

The content of FTY720 in NSC-Exos was determined by HPLC (Agilent 1200, Agilent Technologies). To evaporate solvent, FTY720-NSC-Exos (0.3 mL, contained 20 mg FTY720-NSC-Exos) was heated at 70°C. Then, the same volume of acetonitrile was added, after ultrasound, centrifuging at 24000 g for 10 minutes. The supernatant was filtered with a 0.22 *μ*m syringe filter, and 20 *μ*L aliquots were transferred into HPLC autosampler vials. To measure FTY720 concentration, standard curve of FTY720 was established. Samples were taken at different time points and analyzed using HPLC.

### 2.5. In Vivo

#### 2.5.1. Animals and the SCI Model Establishment

65 Sprague-Dawley (SD) rats (8-week-old, male) were purchased from the Shanghai SLAC Laboratory Animal Co., Ltd. (Shanghai, China). All the rats were allowed to access food and water free and living in standard condition (humidity: 55~70%; room temperature: 23 ± 2°C; light cycle: 12-h light/dark). All animal assays were performed according to the National Institutes of Health Guide for the Care and Use of Laboratory Animals. The animal experiments were in accordance with the guidelines of laboratory animal care and were approved by the Hangzhou Eyong Biotechnological Co., Ltd. Animal Experiment Center (Hangzhou, China).

After 1 week of adaptive feeding, according to previous study, the rats were anesthetized by inhaling 1.5% isoflurane and fixed in the prone position on the heating pad to perform thoracic grade 10 (T10) laminectomy [25]. The spinous procedures of T8 and T11 were clamped to fix the spine, and a laminectomy was performed at the T10 level by Horizons Impactor, causing 50 kd spinal contusion injury to the rats. Rats that only experienced laminectomy and without damage were used as the sham group. After the operation, all rats were placed in an environment of about 38.5°C until they woke up, and the bladder was emptied manually every 8 hours. At the same time, on the first day, all rats were administered with buprenorphine (0.05 mg/kg, ip) every 6 hours to relieve pain.

#### 2.5.2. Experimental Groups

After surgery, 60 rats were divided into 5 groups (*n* = 12/group): sham, model, FTY720 (3 mg/kg) + model, NSC-Exos + model, and FTY720-NSC-Exos + model. After SCI within an hour, rats were treated with NSC-Exos or FTY720-NSC-Exos by tail vein injection (20 *μ*g of NSC-Exos or FTY720-NSC-Exos in 0.3 mL PBS). The FTY720 (3 mg/kg) was administered by gavage, while the sham and model rats were administered 0.3 mL PBS by tail vein injection.

After 48 hours of surgery, the spinal cord was removed from each group of rats (*n* = 6) for immunohistochemistry, HE and TUNEL staining. Three rats were performed to evaluate the spinal cord edema in each group, and the remaining three rats in each group were performed behavioral studies 4 weeks after the surgery.

#### 2.5.3. Rehabilitation of Spinal Cord Function

The Basso, Beattie and Bresnahan (BBB) exercise scale was used to estimate the recovery of hindlimb function after 1, 2, 3, and 4 weeks after injury. Briefly, electromyography was used to detect motor-evoked potentials and evaluate muscle response. After anesthesia, the stimulating electrode was used to stimulate the motor potential on the sciatic nerve and placed the recording electrode into gastrocnemius muscle to record the response of the induced motor potential.

#### 2.5.4. Detection of Spinal Cord Edema

After being anesthetized (100 mg/kg body weight, sodium pentobarbital), T9, T10, and T11 (all about 2 mm) segments were removed from each rat immediately to evaluate the formation of edema. All samples were quantified and placed in a 90°C oven for 72 hours to acquire the dry weight. Edema was calculated as follows: Edema (%) = (wet weight − dry weight)/wet weight∗100 [26].

#### 2.5.5. Tissue Collection

After SCI for 48 hours, rats were anesthetized and perfused with 0.9% saline (containing 50 U/mL heparin) through the endocardium and then perfused with phosphate buffer (containing 4% PFA). A spinal cord segment (10 mm) was cut at the injured site and fixed in 4% PFA for 48 hours at room temperature. Then, the tissues were embedded in paraffin.

#### 2.5.6. Histological Examination

Pathological damages of spinal cord segment tissues were investigated by hematoxylin and eosin (HE) in each group. The spinal cord segment tissues were postfixed and embedded in paraffin, then sectioned into about 4 *μ*m thickness and stained with HE.

#### 2.5.7. Apoptosis Assay

Terminal deoxynucleotidyl transferase dUTP nick end labeling (TUNEL) staining was used to detect the apoptosis during injury by In Situ Cell Death Detection Kit. The sections were deparaffinization and rehydration, then stained in accordance to the instructions of TUNEL kit. The apoptotic activity was detected by optical microscope.

#### 2.5.8. Immunohistochemistry

After embedding in paraffin wax, the tissues were sliced to 4 *μ*m thickness, then incubated in 0.3% H_2_O_2_ for 30 minutes and incubated in 0.1% Triton X-100 for 20 minutes. Next, the sections were incubated with primary antibodies, including anti-Aquaporin-4 (AQP4) antibody (1 : 50; AF5164; Affinity) and anti-claudin-5 antibody (1 : 50; AF5216; Affinity) overnight at 4°C and secondary antibody for 60 min at 37°C. Lastly, the sections were stained with DAB to develop the color and counterstained with hematoxylin.

#### 2.5.9. Western Blotting Assay

The proteins of spinal cord (epicenter ±5 mm) were extracted by radioimmunoprecipitation assay (RIPA) cold lysis buffer. After loaded onto a SDS-PAGE gel, the proteins were separated by electrophoresis and transferred to a polyvinylidene difluoride (PVDF) membrane (GE Healthcare Life, USA). Then, 5% dried skim milk was added to block the nonspecific binding sites of membranes at 37°C for 1.5 hours. After that, the membranes were incubated with dilute solution of antibodies at 4°C overnight. After being washed, secondary antibody were added and incubated with the membranes at 37°C for 1 hour. The protein expression was then tested by enhanced chemiluminescence (ECL) (Solarbio, Beijing, China). The primary antibodies including *β*-actin (1 : 1000; 3700S; CST), anti-Bax antibody (1 : 1000; ab32503; Abcam), and anti-Bcl-2 antibody (1 : 2000; ab182858; Abcam).

### 2.6. In Vitro

#### 2.6.1. Culture and Identification of Spinal Cord Microvascular Endothelial Cells (SCMECs)

Five rats were anesthetized, and the spinal cords were removed. Then, the microvessel fragments were isolated according to previous study [27] and cultured in DMEM medium which contain 10% FBS, 100 *μ*g/mL streptomycin, 100 U/mL penicillin, and 4 *μ*g/mL puromycin. The medium was replaced by the same medium without puromycin after being incubated for 48 hours, then changed the medium every 2 days. When cells reached 80-90% confluence, they were used for the further experiments. Von Willebrand Factor (vWF, 1 : 250; ab154193; Abcam) was performed to detect the contamination of endothelial cell by immunocytochemistry [28].

#### 2.6.2. Establishment of Cell Hypoxic Model

SCMECs were transferred into 6-well plates. When the cells reached 90%, the mediums were changed. SCMECs were divided into five groups: (1) control: SCMECs were cultured in a normal incubator. (2) Hypoxic: SCMECs were incubated in a hypoxic incubator. (3) Hypoxic+FTY720: SCMECs were cultured in the medium which contained 1 mM FTY720 in hypoxia incubator. (4) Hypoxic+NSC-Exos: SCMECs were cultured in the medium which contained 20 *μ*g/mL NSC-Exos. (5) Hypoxic+FTY720-NSC-Exos: SCMECs were cultured in the medium which contained 20 *μ*g/mL FTY720-NSC-Exos (NSC-Exos containing 1 mM FTY720) in hypoxia incubator. Each group was cultured for 6 h [29].

#### 2.6.3. Endothelial Permeability Measurement

The flux of FITC-dextran across the endothelial monolayer was measured by Transwell system to assess the paracellular permeability. Briefly, we put FITC-conjugated dextran in the upper chamber of the Transwell system. Then, aliquots (10 *μ*L) got out from the lower chamber, and the medium was changed at 0, 5, 15, 30, 60, or 90 min, respectively. Finally, the fluorescence of each group was measured.

#### 2.6.4. Western Blotting

The proteins of SCMECs in each group were also extracted using radioimmunoprecipitation assay (RIPA) cold lysis buffer. Specific operational procedures and primary antibodies were in accordance with that described above. The primary antibodies include *β*-actin (1 : 1000; 3700S; CST), anti-PTEN antibody (1 : 1000; ab267787; Abcam), phospho-pan-AKT1/2/3 (Ser473) antibody (1 : 500; ab38449; Affinity), and anti-ZO1 tight junction protein antibody (1 : 1000; ab96587; Abcam).

### 2.7. Statistical Analysis

The statistical analyses of all the data in this study were detected by SPSS 16.0 (IBM, USA). The results are expressed as ^−^*χ* ± *s*. Group comparisons were investigated by Student's *t*-test or the Kruskal-Wallis *H* test according to variance homogeneity and inconsistency. In each experiment, *P* < 0.05 was seen as statistical significance.

## 3. Results

### 3.1. Characterization of NSC-Exos

**Figure 1:**
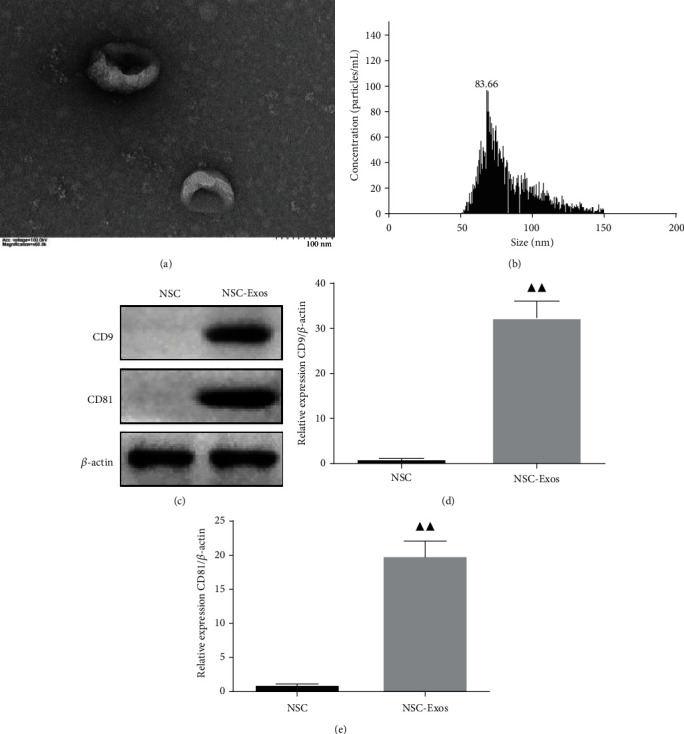
Identification of the neural stem cell exosomes (NSCs-Exos). (a) Morphology of NSCs-Exos was observed by TEM. (b) Nanoparticle size distribution was analyzed by nanoparticle-tracking analysis (NTA). (c) Representative immunoblots of exosomes CD9 and CD81 in different treatment groups. (d, e) Quantitative densitometric analysis of CD9 and CD81 on the exosomes. ^▲^*P* < 0.05, ^▲▲^*P* < 0.01 vs. NSC.

NSCs-Exos were investigated by TEM, NTA, and Western blotting. There were spherical vesicles in NSCs-Exos, which were typically cup-shaped (Figure 1(a)). According to the NTA result, the distribution of NSCs-Exos diameter size were ranged from 30 to 150 nm and showed a relatively normal distribution (Figure 1(b)). Western blotting results showed that CD9 and CD81 were positive in NSCs-Exos and further confirmed the exosomes (Figures 1(c) and 1(d)).

### 3.2. The Concentration of FTY720 in FTY720-NSC-Exos

**Figure 2:**
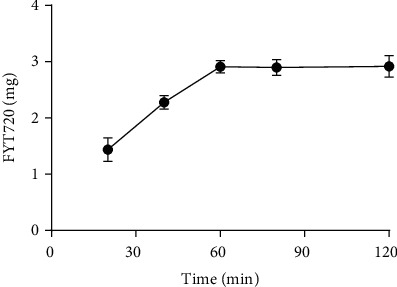
Concentration of FTY720 in FTY720-NSC-Exos with the development of cocultured time.

According to HPLC result, with the time of culturing increased, the concentration of FTY720 was increased. When FTY720 and NSC-Exos cocultured for 60 min, the concentration of FTY720 reached maximum (2.91 ± 0.11 mg) and no longer increased over time (Figure 2). Then, the FTY720-NSC-Exos was used for further study.

### 3.3. In Vivo

#### 3.3.1. FTY720-NSC-Exos Ameliorated Motor Function of SCI Rats

**Figure 3:**
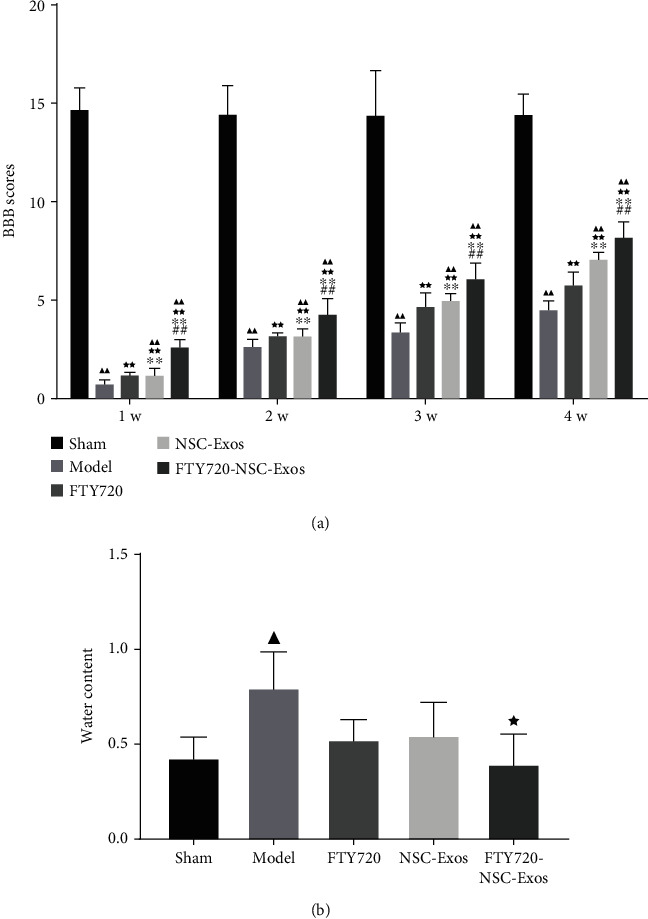
FTY720-NSC-Exos repaired the hindlimb dysfunction and reduced edema in SCI model rats. (a) Basso, Beattie, and Bresnahan (BBB) scores was performed to access the function of hindlimb recovery from 1 to 4 weeks in each group (^−^*χ* ± *s*, *n* = 3). (b) The content of water in spinal cord of each group rats (^−^*χ* ± *s*, *n* = 3). ^▲^*P* < 0.05, ^▲▲^*P* < 0.01 vs. sham, ^★^*P* < 0.05, ^★★^*P* < 0.01 vs. model, ^∗^*P* < 0.05, ^∗∗^*P* < 0.01 vs. FTY720, ^#^*P* < 0.05, ^##^*P* < 0.01 vs. NSCs-Exos.

In order to detect the ameliorated effect of FTY720-NSC-Exos on locomotion of SCI rats, the BBB experiment was performed every week and last 4 weeks in all groups. Compared with the model rats, FTY720-NSC-Exos treatment markedly ameliorated the locomotor function of the hind limbs after SCI 4 weeks (*P* < 0.01). At the same time, the improvement of FTY720-NSC-Exos was better than FTY720 and NSC-Exos (*P* < 0.01). These results revealed that FTY720-NSC-Exos can ameliorate the movement of SCI rats (Figure 3(a)).

#### 3.3.2. FTY720-NSC-Exos Prevented the Formation of Edema

The effect of FTY720-NSC-Exos on the edema formation was evaluated. As shown in Figure 3(b), after SCI, the edema formation was significantly increased compared with sham group. FTY720, NSC-Exos, and FTY720-NSC-Exos significantly inhibited the accumulation of water on the injury site. Similarly, the inhibition of FTY720-NSC-Exos on the edema formation was better than other groups. These results suggested that FTY720-NSC-Exos could protect against edema formation.

#### 3.3.3. FTY720-NSC-Exos Reduced SCI Lesion

**Figure 4:**
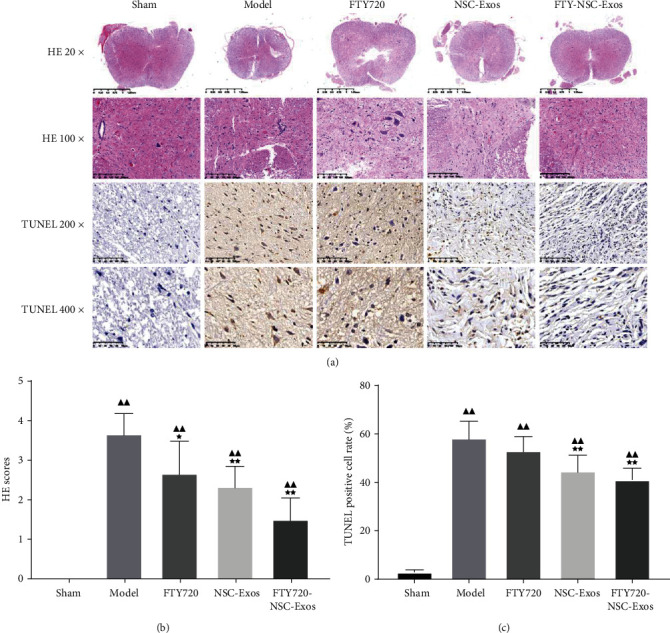
FTY720-NSC-Exos restored histological abnormalities and alleviated damages in spinal cord. (a) Representative microphotographs of HE (original magnification 20x, 100x) and TUNEL staining in each group (original magnification 200x, 400x). (b) Semiquantitative estimate of the histological lesions. (c) Quantitative analysis of the neuronal apoptosis in the lesions site of the spinal cord (^−^*χ* ± *s*, *n* = 6), ^▲^*P* < 0.05, ^▲▲^*P* < 0.01 vs. sham, ^★^*P* < 0.05, ^★★^*P* < 0.01 vs. model.

HE assay result implied the structure of spinal cord in sham group rats was complete, neural cell morphology was normal, and no inflammatory cell infiltration was found. However, the morphology and tissue structure of spinal cord was incomplete and disordered, and the cell nuclear was split or even disappeared in SCI group. Meanwhile, a large number of inflammatory cell infiltration was observed and the interspace of cells and vascular was enlarged. After treated with FTY720, NSC-Exos, and FTY720-NSC-Exos, the inflammatory cell infiltration was less, and the tissue structure was more complete. The ameliorate effect on inflammation between the FTY720 and NSC-Exos had no significant differences. Particularly, rats which received FTY720-NSC-Exos showed a lower level of SCI lesion than other rats (Figures 4(a) and 4(b)).

#### 3.3.4. FTY720-NSC-Exos Attenuated Cell Apoptosis

The number of apoptosis neuronal cells in the injury site of the spinal cord was assessed by TUNEL assay (Figures 4(a) and 4(c)). Compared with the model rats, the number of TUNEL-positive cells decreased obviously after treated with FTY720, NSC-Exos, and FTY720-NSC-Exos (*P* < 0.01). Meanwhile, the apoptotic index in the FTY720-NSC-Exos groups was markedly lower than that of the other two groups (*P* < 0.01).

#### 3.3.5. The Effect of FTY720-NSC-Exos on the Edema-Related Proteins

**Figure 5:**
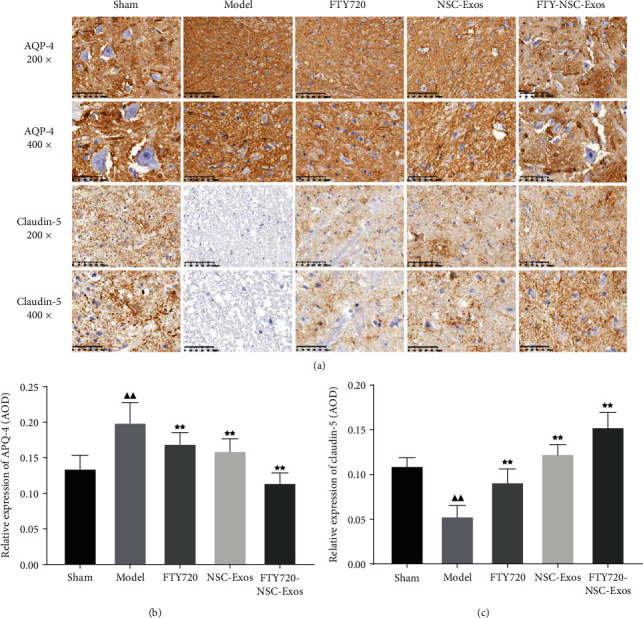
The expression level of AQP-4 and claudin-5 was investigated by immunohistochemical in each group rats. (a) Representative microphotographs of AQP-4 and claudin-5 (original magnification 200x, 400x). (b) Quantitative analysis of AQP-4 and claudin-5 protein expression (^−^*χ* ± *s*, *n* = 6), ^▲^*P* < 0.05, ^▲▲^*P* < 0.01 vs. sham, ^★^*P* < 0.05, ^★★^*P* < 0.01 vs. model, ^∗^*P* < 0.05, ^∗∗^*P* < 0.01 vs. FTY720, ^#^*P* < 0.05, ^##^*P* < 0.01 vs. NSCs-Exos.

Aquaporins acts as a key role in keeping the spinal cord water balance. The effect of exosomes on the expression of AQP4 and claudin-5 was detected by immunohistochemistry. The expression of claudin-5 was markedly decreased, while AQP4 was increased (*P* < 0.01). However, FTY720, NSC-Exos, and FTY720-NSC-Exos increased the expression of claudin-5. Meanwhile, the expression of AQP4 was decreased obviously (*P* < 0.01, Figure 5).

#### 3.3.6. The Effect of FTY720-NSC-Exos on the Apoptosis-Related Proteins

**Figure 6:**
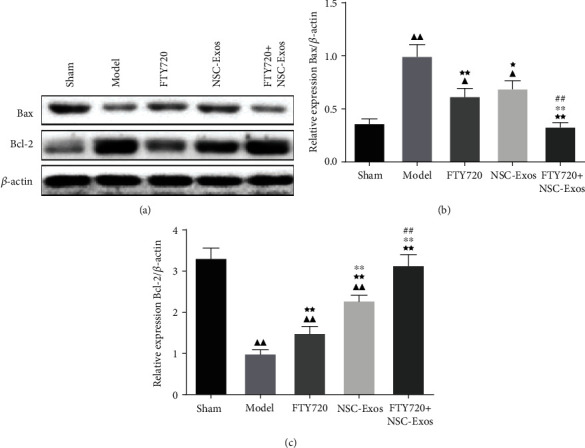
(a–c) The expressions of apoptotic-related proteins in each groups were determined by WB (^−^*χ* ± *s*, *n* = 6), ^▲^*P* < 0.05, ^▲▲^*P* < 0.01 vs. sham, ^★^*P* < 0.05, ^★★^*P* < 0.01 vs. model, ^∗^*P* < 0.05, ^∗∗^*P* < 0.01 vs. FTY720, ^#^*P* < 0.05, ^##^*P* < 0.01 vs. NSCs-Exos.

In order to detect the effect of FTY720-NSC-Exos on the apoptosis-related proteins, Western blotting was performed. The protein expression of Bax was markedly downregulated, and the protein expression of Bcl-2 was markedly upregulated. However, compared to the model group, FTY720, NSC-Exos, and FTY720-NSC-Exos attenuated the expression of Bax and upregulated the expression of Bcl-2 (*P* < 0.01). What is more, the inhibitory effect of FTY720-NSC-Exos on apoptosis was better than FTY720 and NSC-Exos (*P* < 0.01) (Figure 6).

### 3.4. In Vitro

#### 3.4.1. Identification of SCMECs

**Figure 7:**
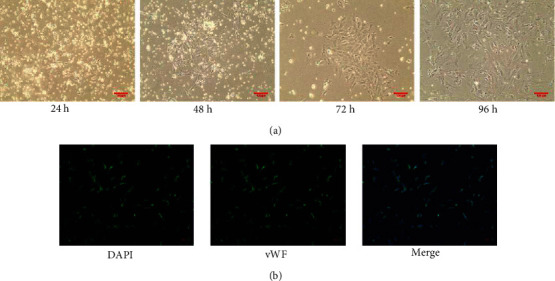
Identification of SCMECs. (a) Morphology photographs of SCMECs (original magnification 100x). (b) Expression of vWF antibodies in SCMECs (original magnification 200x) (^−^*χ* ± *s*, *n* = 6).

The morphology of microvessel segments were typically beaded or branched, and SCMECs crawled out of the microvessel segments after 48 hours. Then, the SCMECs are with a typical “pebble” shape and adherent growth. After 72 hours, the cells gradually increased and were identified by labeled with vWF (Figure 7(a)).  Figure 5(b) showed that vWF had a high expression in SCMECs.

#### 3.4.2. FTY720-NSC-Exos Protected SCMECs under Hypoxic Conditions

**Figure 8:**
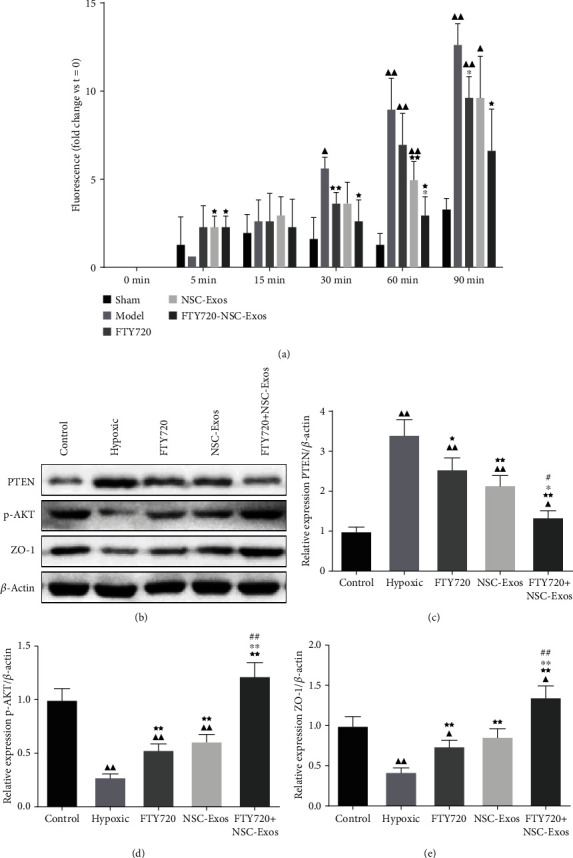
FTY720-NSC-Exos protected SCMECs under hypoxic conditions. (a) The permeability of the SCMECs was detected by Transwell experiment. (b–e) The protein level of PTEN, p-AKT, and ZO-1 in each groups (^−^*χ* ± *s*, *n* = 6), ^▲^*P* < 0.05, ^▲▲^*P* < 0.01 vs. control, ^★^*P* < 0.05, ^★★^*P* < 0.01 vs. hypoxic, ^∗^*P* < 0.05, ^∗∗^*P* < 0.01 vs. FTY720, ^#^*P* < 0.05, ^##^*P* < 0.01 vs. NSCs-Exos.

Transwell experiment was performed to detect the permeability of SCMECs. The permeability of the SCMECs was highly improved under hypoxic circumstance. Interestingly, the permeability induced by hypoxia was decreased after treated with FTY720, NSC-Exos, and FTY720-NSC-Exos (*P* < 0.05). These results were consistent with Western blotting results; FTY720, NSC-Exos, and FTY720-NSC-Exos can downregulate the expression of PTEN and upregulate the expression of p-Akt and ZO-1 in SCMECs under hypoxic circumstance (Figure 8). Moreover, the improvement of FTY720-NSC-Exos on the permeability was better than others, which indicates that FTY720-NSC-Exos treatment can protect SCMECs under hypoxic conditions.

## 4. Discussion

We investigated the improvement of FTY720-NSC-Exos on locomotors in SCI rats and the mechanism of FTY720-NSC-Exos in improving secondary degeneration after SCI. FTY720 is the nonselective S1P (S1P_1_, S1P_3_, S1P_4_, and S1P_5_) receptor agonist and is phosphorylated by sphingosine kinase [30]. At present, S1P1 receptor-mediated immunosuppression is the common systemic effect of FTY720 administration [31]. The positive effectivity of FTY720 has been demonstrated in multifarious autoimmune disorders and allograft survival animal models [32–34]. It was believed that immunity is the main biological activity of these diseases, but our study demonstrated the nonimmunity impact of FTY720 in the SCI treatment.

At the same time, stem cell transplantation has shown a good application prospect in the treatment of SCI [35]. In the past, homing, differentiation, and replacement of injured cells were seen as the repair mechanism of stem cells [36]. However, the repairment of extracellular vesicles secreted by transplanted cells to SCI was attracted more attention nowadays [37]. The exosomes of NSCs play a consistent effect with NSC transplantation and avoid the limitations of direct stem cell transplantation. Firstly, the NSCs-Exos were derived from NSCs and with a diameter range from 30 to 150 nm by using NTA analysis in this study. At the same time, surface markers CD9 and CD81 were expressed on the surface of NSCs-Exos. And the morphology of NSCs-Exos was confirmed by TEM. After that, we prepared NSCs-Exos containing FTY720 and conducted a series of experiments to investigate the therapeutic effect in the SCI model *in vivo* and *in vitro*.

A range of complex pathophysiological changes, such as hemorrhage, ischemia, edema, damage of the blood-spinal barrier, and obstacles to microhemodynamics were caused by SCI [38]. These changes further promote cell apoptosis, necrosis, inflammatory cells infiltration, and the reconstruction of inhibitory functional synapses, which in turn affect functional recovery [39]. Tissue edema is a common symptom in SCI and caused by raised permeability of the blood spinal cord barrier (BSCB) [40]. The permeability changes of BSCB increased the exchange of harmful factors in tissue and blood and further induced cell death and permanent neurological dysfunction [41]. Claudin-5 is mainly expressed of endothelial cells and acts a key role in BSCB [42]. During SCI, alterations of its distribution and expression affect the changes of BSCB permeability [43]. Reducing the formation of edema after traumatic SCI is beneficial to the functional recovery of acute and chronic stage after damage [44]. In this study, we observed that FYT720-NSCS-Exos markedly reduced the formation of edema and increased the expression of Claudin-5 after SCI. Meanwhile, AQP4 acts a key role in angioedema and cytotoxic edema and is essential for the removal of angioedema [45]. When the expression of AQP4 was high, the liquid was entered into cells and caused cytotoxic edema. Here, the expression of AQP4 in the damage location was raised. It might be the increased expression of AQP4 promotes water entered into spinal cord parenchyma from the vasculature and increases the spinal cord swelling after injury. After treated with FYT720-NSCS-Exos, the expression of AQP4 was significantly reduced. This suggested that FYT720-NSCS-Exos can alleviate spinal cord edema by regulating the expression of claudin-5 and AQP4 proteins during SCI.

Apoptosis is related to many central nervous system diseases and affects the functional recovery after SCI [46]. Meanwhile, Bax and Bcl-2 are key cell apoptosis-related components. TUNEL assay showed that FYT720-NSCS-Exos treatment can reduce the apoptosis of neuronal cells, which was consistent with Western blotting assay. The protein level of Bax was markedly decreased, while the level of Bcl-2 was increased. These assays all implied that FYT720-NSCS-Exos treatment could protect neuronal cells from apoptosis induced by SCI. In addition, the administration of FYT720-NSCS-Exos also improved the morphology of neuronal cells, reduced inflammatory infiltration, and further improved motor behaviour of the hind limbs.

In order to further estimate the mechanism of barrier protection, the permeability of endothelial cells was evaluated by the Transwell assay in an *in vitro* hypoxia model. The result showed that FYT720-NSCS-Exos can availably protect the endothelial barrier and promote the expression of ZO-1 under the hypoxic circumstance. PI3K/AKT pathway is a regulator of apoptosis and survival, and PTEN is considered to be a negative regulator of the PI3K/AKT pathway [47]. p-Akt is a key protein in PI3K/AKT pathway and could inhibit cell apoptosis and improve cell survival [48]. Here, we confirmed that FYT720-NSCS-Exos downregulated the expression of PTEN and promoted the expression of p-Akt in SCMECs. It is suggested that FYT720-NSCS-Exos could reduce cell apoptosis by regulating the PTEN/AKT signal pathway.

In conclusion, we demonstrated that FTY720-NSCs-Exos treatment after SCI can promote the morphological of neurons, thereby improving hindlimb motor behavior. At the same time, FTY720-NSCs-Exos treatment could improve endothelial function and alleviate cell apoptotic and spinal cord edema, thereby ameliorating functional and behavioral after SCI by regulating the PTEN/AKT signal pathway. Meanwhile, the protection of the endothelial cells under hypoxia circumstance might be one of its potential mechanisms. These findings indicated that FTY720-NSCs-Exos is a promising therapy for SCI.

## Data Availability

All data generated or analyzed during this study are included in this article.

## References

[1] Kumar R., Lim J., Mekary R. A. (2018). Traumatic spinal injury: global epidemiology and worldwide volume. *World Neurosurgery*.

[2] Lattard A., Poulen G., Bartolami S., Gerber Y. N., Perrin F. E. (2021). Negative impact of sigma-1 receptor agonist treatment on tissue integrity and motor function following spinal cord injury. *Frontiers in Pharmacology.*.

[3] Yuan X., Wu Q., Wang P. (2019). Exosomes derived from pericytes improve microcirculation and protect blood-spinal cord barrier after spinal cord injury in mice. *Frontiers in Neuroscience*.

[4] Zhang X., Liu X. D., Xian Y. F., Zhang F., Lin Z. X. (2019). Berberine enhances survival and axonal regeneration of motoneurons following spinal root avulsion and re-implantation in rats. *Free Radical Biology and Medicine*.

[5] Penney J., Tsai L. H. (2015). JAKMIP1: translating the message for social behavior. *Neuron*.

[6] Fukuoka T., Kato A., Hirano M., Natsume A. (2021). Neurod4 converts endogenous neural stem cells to neurons with synaptic formation after spinal cord injury. *IScience*.

[7] Deng M., Xie P., Chen Z. (2021). Mash-1 modified neural stem cells transplantation promotes neural stem cells differentiation into neurons to further improve locomotor functional recovery in spinal cord injury rats. *Gene*.

[8] Zhang L., Fan C., Hao W. (2021). Nscs migration promoted and drug delivered exosomes-collagen scaffold via a bio-specific peptide for one-step spinal cord injury repair. *Advanced Healthcare Materials*.

[9] Jeong J. O., Han J. W., Kim J. M. (2011). Malignant tumor formation after transplantation of short-term cultured bone marrow mesenchymal stem cells in experimental myocardial infarction and diabetic neuropathy. *Circulation Research*.

[10] Koprivec S., Novak M., Bernik S., Majdič G. (2021). Treatment of cranial cruciate ligament injuries in dogs using a combination of tibial tuberosity advancement procedure and autologous mesenchymal stem cells/multipotent mesenchymal stromal cells - a pilot study. *Acta Veterinaria Hungarica*.

[11] Nargesi A. A., Lerman L. O., Eirin A. (2017). Mesenchymal stem cell-derived extracellular vesicles for kidney repair: current status and looming challenges. *Stem Cell Research & Therapy*.

[12] Keshtkar S., Azarpira N., Ghahremani M. H. (2018). Mesenchymal stem cell-derived extracellular vesicles: novel frontiers in regenerative medicine. *Stem Cell Research & Therapy.*.

[13] Azmi A. S., Bao B., Sarkar F. H. (2013). Exosomes in cancer development, metastasis, and drug resistance: a comprehensive review. *Cancer Metastasis Reviews*.

[14] Tofaris G. K. (2017). A critical assessment of exosomes in the pathogenesis and stratification of Parkinson’s disease. *Journal of Parkinson's Disease*.

[15] Li W. Y., Zhu Q. B., Jin L. Y., Hu X. Y. (2021). Exosomes derived from human induced pluripotent stem cell-derived neural progenitor cells protect neuronal function under ischemic conditions. *Neural Regeneration Research*.

[16] Harrell C. R., Volarevic A., Djonov V., Volarevic V. (2021). Mesenchymal stem cell-derived exosomes as new remedy for the treatment of neurocognitive disorders. *International Journal of Molecular Sciences.*.

[17] Bowers D. T., Olingy C. E., Chhabra P. (2018). An engineered macroencapsulation membrane releasing fty720 to precondition pancreatic islet transplantation. *Journal of Biomedical Materials Research. Part B, Applied Biomaterials*.

[18] Norimatsu Y., Ohmori T., Kimura A. (2012). FTY720 improves functional recovery after spinal cord injury by primarily nonimmunomodulatory mechanisms. *The American Journal of Pathology*.

[19] Yamazaki K., Kawabori M., Seki T. (2020). Fty720 attenuates neuropathic pain after spinal cord injury by decreasing systemic and local inflammation in a rat spinal cord compression model. *Journal of Neurotrauma*.

[20] Sim-Selley L. J., Wilkerson J. L., Burston J. J. (2018). Differential tolerance to fty720-induced antinociception in acute thermal and nerve injury mouse pain models: role of sphingosine-1-phosphate receptor adaptation. *Journal of Pharmacology & Experimental Therapeutics*.

[21] Xin H., Li Y., Buller B. (2012). Exosome-mediated transfer of miR-133b from multipotent mesenchymal stromal cells to neural cells contributes to neurite outgrowth. *Stem Cells*.

[22] Villarroya-Beltri C., Gutiérrez-Vázquez C., Sánchez-Madrid F., Mittelbrunn M. (2013). Analysis of microRNA and protein transfer by exosomes during an immune synapse. *Methods in Molecular Biology*.

[23] Dissanayake K., Midekessa G., Lttekivi F., Fazeli A. (2021). Measurement of the size and concentration and zeta potential of extracellular vesicles using nanoparticle tracking analyzer. *Methods in Molecular Biology*.

[24] Carnell-Morris P., Tannetta D., Siupa A., Hole P., Dragovic R. (2017). Analysis of extracellular vesicles using fuorescence nanoparticle tracking analysis. *Methods in Molecular Biology*.

[25] Yuan X. C., Wang P., Li H. W. (2017). Effects of melatonin on spinal cord injury-induced oxidative damage in mice testis. *Andrologia*.

[26] Winkler T., Sharma H. S., Stlberg E., Badgaiyan R. D. (2000). Neurotrophic factors attenuate alterations in spinal cord evoked potentials and edema formation following trauma to the rat spinal cord. *Acta Neurochirurgica. Supplement*.

[27] Yuan X., Wu Q., Liu X., Zhang H., Xiu R. (2018). Transcriptomic profile analysis of brain microvascular pericytes in spontaneously hypertensive rats by RNA-Seq. *American Journal of Translational Research*.

[28] Wu Q., Jing Y., Yuan X. (2015). The distinct abilities of tube-formation and migration between brain and spinal cord microvascular pericytes in rats. *Clinical Hemorheology and Microcirculation*.

[29] Nohda K., Nakatsuka T., Takeda D. (2007). Selective vulnerability to ischemia in the rat spinal cord: a comparison between ventral and dorsal horn neurons. *Spine*.

[30] Brinkmann V., Cyster J. G., Hla T. (2004). FTY720: sphingosine 1-phosphate receptor-1 in the control of lymphocyte egress and endothelial barrier function. *American Journal of Transplantation*.

[31] Mandala S., Hajdu R., Bergstrom J. (2002). Alteration of lymphocyte trafficking by sphingosine-1-phosphate receptor agonists. *Science*.

[32] Aoki M., Kondo A., Matsunaga N., Honda A., Ogawa R. (2020). The immunosuppressant fingolimod (FTY720) for the treatment of mechanical force-induced abnormal scars. *Journal of Immunology Research*.

[33] Guo X. D., Ji J., Xue T. F., Sun Y. Q., Sun X. L. (2020). Fty720 exerts anti-glioma effects by regulating the glioma microenvironment through increased cxcr4 internalization by glioma-associated microglia. *Frontiers in Immunology*.

[34] Perla A. S., Fratini L., Cardoso P. S., CBD F., Roesler R. (2019). Fingolimod (fty720) reduces viability and survival and increases histone h3 acetylation in medulloblastoma cells. *Pediatric Hematology and Oncology*.

[35] Jones I., Novikova L. N., Wiberg M., Novikov L. N. (2021). Human embryonic stem cell-derived neural crest cells promote sprouting and motor recovery following spinal cord injury in adult rats. *Cell Transplantation*.

[36] Sadat-Ali M., Al-Dakheel D. A., Ahmed A., Al-Bayat M. I. (2020). Spinal cord injury regeneration using autologous bone marrow-derived neurocytes and rat embryonic stem cells: a comparative study in rats. *World Journal of Stem Cells*.

[37] Ratajczak M. Z., Jadczyk T., Pêdziwiatr D., Wojakowski W. (2014). New advances in stem cell research: practical implications for regenerative medicine. *Polskie Archiwum Medycyny Wewnętrznej*.

[38] Wang J., Zhao W., Wang X., Yan J. (2021). Enhanced store-operated calcium entry (SOCE) exacerbates motor neurons apoptosis following spinal cord injury. *Gen Physiol Biophys*.

[39] Alilain W. J., Horn K. P., Hu H., Dick T. E., Silver J. J. N. (2011). Functional regeneration of respiratory pathways after spinal cord injury. *Nature*.

[40] Ying X., Xie Q., Li S., Jiang S. (2020). Water treadmill training attenuates blood-spinal cord barrier disruption in rats by promoting angiogenesis and inhibiting matrix metalloproteinase-2/9 expression following spinal cord injury. *Fluids Barriers CNS*.

[41] Fan Z. K., Cao Y., Lv G., Wang Y. S., Guo Z. P. (2013). The effect of cigarette smoke exposure on spinal cord injury in rats. *Journal of Neurotrauma*.

[42] Kong Y., Yang Y., Guan Q. B., Guo L., Han C. Y. (2019). Study on the effect of bfgf combined with antrodia camphorate polysaccharide on the repair of neural function after mechanical spinal cord injury. *Chin J Mod Appl Pharm*.

[43] Liebner S., Czupalla C. J., Wolburg H. (2011). Current concepts of bloodbrain barrier development. *The International Journal of Developmental Biology*.

[44] Fukuda A. M., Adami A., Pop V. (2013). Posttraumatic reduction of edema with aquaporin-4 RNA interference improves acute and chronic functional recovery. *Journal of Cerebral Blood Flow and Metabolism*.

[45] Hubbard J. A., Szu J. I., Yonan J. M., Binder D. K. (2016). Regulation of astrocyte glutamate transporter-1 (GLT1) and aquaporin-4 (AQP4) expression in a model of epilepsy. *Experimental Neurology*.

[46] Zhang J., Cui Z., Feng G. (2015). RBM5 and p53 expression after rat spinal cord injury: implications for neuronal apoptosis. *The International Journal of Biochemistry & Cell Biology*.

[47] Shen S., Zhang M., Ma M., Qu J. (2021). Potential neuroprotective mechanisms of methamphetamine treatment in traumatic brain injury defined by large-scale ionstar-based quantitative proteomics. *International Journal of Molecular Sciences*.

[48] Saurav B., Abdul-Muneer P. M. (2020). Pten blocking stimulates corticospinal and raphespinal axonal regeneration and promotes functional recovery after spinal cord injury. *Journal of Neuropathology & Experimental Neurology*.

